# Determination of leaf carbon isotope discrimination in C4 plants under variable N and water supply

**DOI:** 10.1038/s41598-017-00498-w

**Published:** 2017-03-23

**Authors:** Hao Yang, Qiang Yu, Wen-ping Sheng, Sheng-gong Li, Jing Tian

**Affiliations:** 1 Key Laboratory of Ecosystem Observation and Modeling, Institute of Geographic Sciences and Natural Resources Research, Chinese Academy of Sciences, Beijing, 100101 China; 2 National Hulunber Grassland Ecosystem Observation and Research Station, Institute of Agricultural Resources and Regional Planning, Chinese Academy of Agricultural Sciences, Beijing, 100081 China

## Abstract

Understanding the mechanisms underlying variations in carbon isotope discrimination (*Δ*) in C4 plants is critical for predicting the C3/C4 ratio in C3/C4 mixed grassland. The value of *Δ* is determined by bundle sheath leakiness (*Ф*) and the ratio of intercellular to ambient CO_2_ concentration (*C*_*i*_/*C*_*a*_). Leaf nitrogen concentration (*N*_*leaf*_) is considered a driver of *Δ* in C4 plants. However, little is known about how *N*_*leaf*_ affects *Ф* and *C*_*i*_/*C*_*a*_, and subsequently *Δ*. Here leaf carbon isotope composition, *N*_*leaf*_, *Ф*, and leaf gas exchange were measured in *Cleistogenes squarrosa*, a dominant C4 species in the Inner Mongolia grassland. *Δ* remained relatively stable under variable N and water supply. Higher N supply and lower water supply increased *N*_*leaf*_, stimulated photosynthesis and further decreased *C*_*i*_/*C*_*a*_. High N supply increased *Ф*, which responded weakly to water supply. *N*_*leaf*_ exerted similar effects on *C*_*i*_/*C*_*a*_ and on *Ф* in the field and pot experiments. Pooling all the data, *N*_*leaf*_ explained 73% of the variation in *C*_*i*_/*C*_*a*_. Overall, both *Ф* and *C*_*i*_/*C*_*a*_ determined *Δ*; however, the contribution of *Ф* was stronger. *N*_*leaf*_ influenced *Δ* primarily though *C*_*i*_/*C*_*a*_, rather than *Ф*. *Ф* should be considered in estimating *Δ* of C4 endmember.

## Introduction

The carbon isotope discrimination (*Δ*) of C4 plants, C3 plants, and bulk samples (e. g., bulk vegetation, soil organic matter, wool, and horn) are widely used to calculate the C3/C4 ratio based on a two-member mixed model^1–3^. A single mean value of C4 end-member is usually used because it is weak responsive to environmental variables. However, this view should be changed based on a number of studies. The *Δ* of C4 plants is closely related to the environmental variables of precipitation^4, 5^, atmospheric CO_2_ concentration^6, 7^ and human disturbance, such as grazing^8^, as these factors can affect the ecophysiological responses of C4 plants. Further, leaf nitrogen concentration (*N*_*leaf*_) is considered a possible physiological driver of the variation in *Δ* ^8^. A better understanding of the mechanisms underlying the influence of *N*_*leaf*_ on *Δ* is fundamental to predict the variation in *Δ* in C4 plants, especially in the light of the doubled availability of reactive global nitrogen over the last 50 years^9^.

The *Δ* in C4 plants is influenced by many factors, such as isotope effects during diffusion of CO_2_ through stomatal pore and cell walls, fixation of bicarbonate by phosphenolpyruvate carboxylase (PEPC) in mesophyll cells, fixation of CO_2_ by Rubisco in bundle sheath cells, and leakage of CO_2_ from bundle sheath cells to mesophyll cells^10, 11^. Farquhar *et al.* ^11^ proposed a simplified model, which was widely used to analyze the relationships between environment factors and *Δ*. The *Δ* in C_4_ plants depends on the ratio of intercellular to ambient CO_2_ concentration (*C*_*i*_/*C*_*a*_) and bundle sheath leakiness (*Ф*, the proportion of C fixed by PEP carboxylation, which subsequently leaks out of the bundle sheath):

1 $$\Delta =a+({b}_{4}+{b}_{3}\cdot \varphi -a)\cdot {C}_{i}/{C}_{a}$$

where *a* is the discrimination of ^13^C during diffusion of CO_2_ through stomata (4.4‰), *b*_*3*_ is the fixation by Rubisco 27‰ for C4 plants^12^, and *b*_*4*_ is the hydration of CO_2_ to ${{\rm{HCO}}}_{3}^{-}$ and fixation by PEP carboxylase (PEPC) 5.7‰ depending on the temperature^13^.

In equation (1), Δ varies with *Ф* and *C*_*i*_/*C*_*a*_. *Ф* is an important C4 photosynthesis parameter and is mostly affected by the CO_2_ concentration gradient between bundle sheath and mesophyll cells, and thus by factors influencing the activity ratio of Rubisco to PEPC^11^. A high value of *Ф* represents inefficiency in the CO_2_ concentration processes and increases the quantum requirement in C4 photosynthesis^11, 14^. Variation in *N*_*leaf*_ can influence the allotment of nitrogen to Rubisco and PEPC. Under nitrogen-rich conditions, *N*_*leaf*_ is high and PEPC activity can increase to a greater extent than Rubisco, resulting in a high CO_2_ concentration gradient between bundle sheath and mesophyll cells and then a high *Ф* value^15, 16^. However, different results were also reported in several studies^17–19^ and thus the relationship between *N*_*leaf*_ and *Ф* might be species-specific. In terms of *C*_*i*_/*C*_*a*_, increasing *N*_*leaf*_ improves the allotment of nitrogen to photosynthetic enzymes and further decreases the *C*_*i*_/*C*_*a*_ through stimulating the photosynthetic capacity of the plant^20, 21^. *C*_*i*_/*C*_*a*_ also depends on stomatal conductance, which is influenced by the vapor pressure deficit and leaf water potential affected by available water in the soil^22^. Low soil water availability could decrease *C*_*i*_/*C*_*a*_ by closing stomata and influence the nitrogen uptake by roots. Hence, the effects of *N*_*leaf*_ and soil water on *C*_*i*_/*C*_*a*_ might interact and be difficult to distinguish. Thus, the mechanism of how *N*_*leaf*_ influence both *Ф* and *C*_*i*_/*C*_*a*_, and in turn *Δ* is still poorly understood.

*Cleistogenes squarrosa* (Trin. ex Ledeb.) Keng is a dominant C_4_ plants that occurs across a wide range of habitats, such as meadow steppe, typical steppe, desert steppe, and sand dune ecosystems in the semi-arid Inner Mongolian grassland. As a NAD-ME subtype lacking a suberized lamella^23^, *C. squarrosa* is more sensitive to N and water supply. In this study, we measured *Δ* and photosynthetic gas exchange and obtained *Ф* under increased N supply and limited soil water (W). The effects of *N*_*leaf*_ on *Ф* and *C*_*i*_/*C*_*a*_ and the determination of *Ф* and *C*_*i*_/*C*_*a*_ on *Δ* were assessed. Specifically, the following three questions were addressed: Firstly, how do different N and W supply affect *Δ*, *N*_*leaf*_, and related gas exchange? Secondly, how doed *N*_*leaf*_ affect *Ф* and *C*_*i*_/*C*_*a*_? Thirdly, which is the major factor affecting *Δ* − *Ф* or *C*_*i*_/*C*_*a*_?

## Results

### Effects of N and water supply on *Δ* and related parameters in the pot experiment

In the pot experiment, the values of *Δ* varied from 5.14‰ to 6.85‰ (SD = 0.37‰). N and W supply had no significant effect on *Δ*, although *P* = 0.06 for N supply effect (Table 1). High N supply and low W supply enhanced *N*_*leaf*_ and decreased *C*_*i*_/*C*_*a*_, and the N × W interaction was significant for *C*_*i*_/*C*_*a*_. *C*_*i*_/*C*_*a*_ was lower, in spite of higher *g*_*s*_, under low W supply. *Ф* increased with N supply and showed no response to water supply while the N × W interaction was significant. *Ф* and *C*_*i*_/*C*_*a*_ varied between 0.43 and 0.77 (average = 0.51, SD = 0.07) and between 0.18 and 0.67 (average = 0.43, SD = 0.17), respectively.

**Table 1:** Statistical significance of photosynthetic rate (*A*), stomatal conductance (*g*_*s*_), leaf nitrogen content (*N*_*leaf*_), the ratio of intercellular to ambient CO_2_ concentration (*C*_*i*_/*C*_*a*_), carbon isotope discrimination (*Δ*), and bundle sheath leakiness (*Ф*) in *Cleistogenes squarrosa* responses to N and water (W) supply in the pot experiment.

	Source of variation^a^					
	*A*	*g*_*s*_	*N*_*leaf*_	*C*_*i*_/*C*_*a*_	*Δ*	*Ф*
N supply	*	ns	**	**	ns	**
W supply	**	**	*	*	ns	ns
N supply × W supply	**	*	ns	**	ns	*

^a^*, **, and ns for *P* < 0.05, *P* < 0.01, and not significant, respectively.

**Figure 1:**
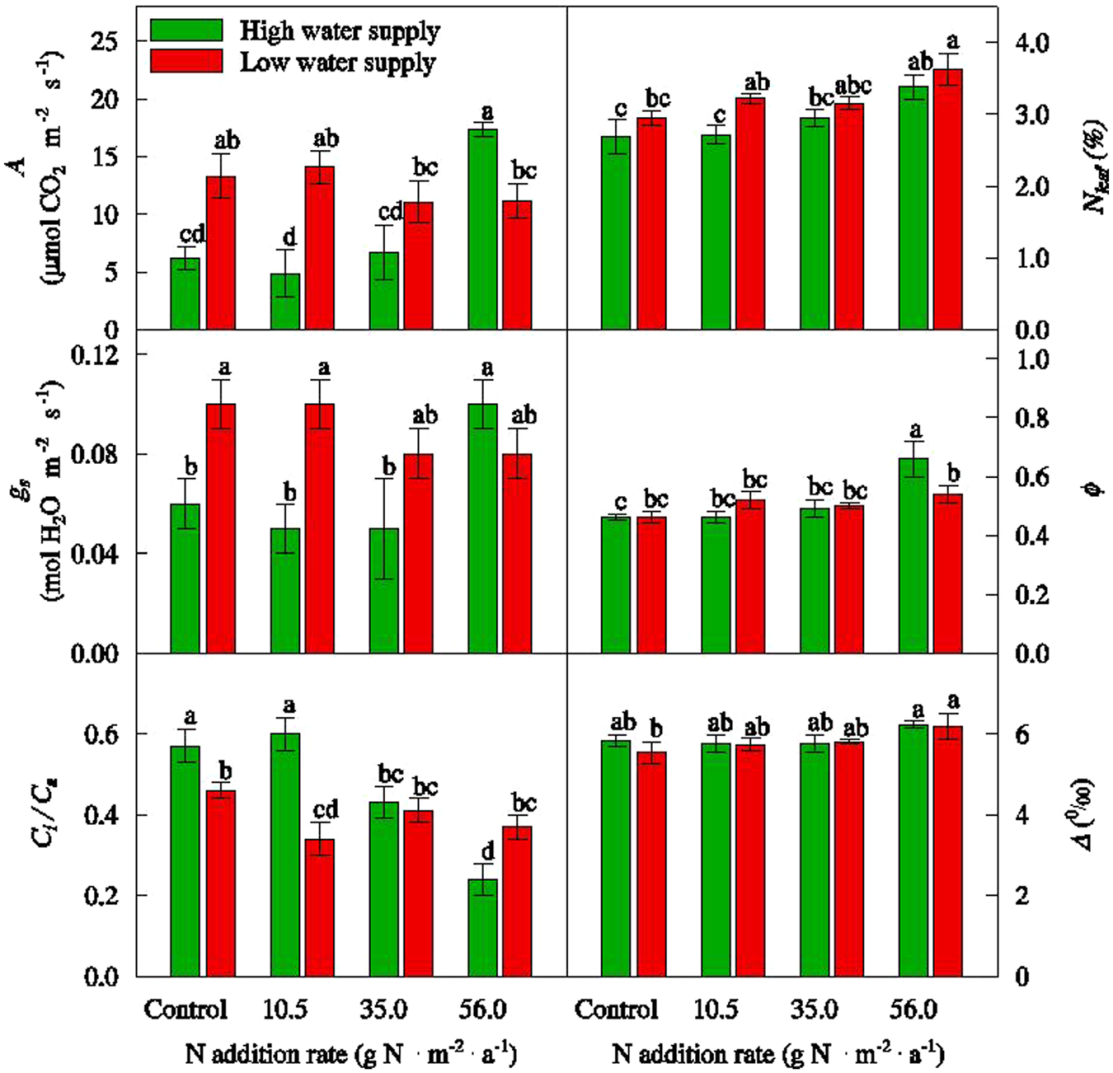
Photosynthetic rate (*A*), stomatal conductance (*g*_*s*_), ratio of internal to ambient CO_2_ partial pressure (*C*_*i*_/*C*_*a*_), leaf nitrogen concentration (*N*_*leaf*_), bundle sheath leakiness (*Ф*), and carbon isotope discrimination (*Δ*) in *Cleistogenes squarrosa* grown under variable N and water supply in the pot experiment. Error bars indicate standard error (N = 4). Different letters indicate significant differences between treatments at *P* = 0.05.

The effects of N supply on *A*, *g*_*s*_, *C*_*i*_/*C*_*a*_, and *N*_*leaf*_ were different under different W supply (Fig. 1). Under high W supply, higher N supply enhanced *N*_*leaf*_ and stimulated *A*. *C*_*i*_/*C*_*a*_ decreased rapidly with more N supply although *g*_*s*_ increased. Compared to no N supply, *A* was three times higher and *C*_*i*_/*C*_*a*_ was three times lower at the N supply of 56.0 g N m^−2^. Under low W supply, N supply had no significant effect on *A*, *g*_*s*_, or *C*_*i*_/*C*_*a*_, except for increasing *N*_*leaf*_. The effects of W supply on *A*, *g*_*s*_, *C*_*i*_/*C*_*a*_, and *N*_*leaf*_ were also different under different N supply (Fig. 1). At no or low N supplies (e.g., 10.5 g N m^−2^), low W supply enhanced *N*_*leaf*_ and *g*_*s*_ and then stimulated *A*, and decreased *C*_*i*_/*C*_*a*_. However, at the N supply of 56.0 g N m^−2^, low W supply decreased *A* and *C*_*i*_/*C*_*a*_ in spite of a slight increase in *N*_*leaf*_.

The responses of *Ф* and *Δ* to N supply were dependent on W supply (Fig. 1). Under high W supply, N supply increased *Ф* and had no effect on *Δ*. *Ф* was highest at the N supply of 56.0 g N m^−2^ and lowest at no N supply. Under low W supply, *Ф* had no response to N supply, while *Δ* was slightly higher at the N supply of 56.0 g N m^−2^ compared to no N supply. In terms of W supply, higher W supply increased *Ф* at the N supply of 56.0 g N m^−2^.

### Effects of N supply on *Δ* and related parameters in the field experiment

In the field experiment, N supply had no significant effect on *Δ* and the related parameters of *A*, *g*_*s*_, *C*_*i*_/*C*_*a*_, *N*_*leaf*_, and *Ф*. However, *N*_*leaf*_ and *Ф* showed trends of increasing, while *C*_*i*_/*C*_*a*_ showed a decreasing trend, which was similar to the results of the pot experiment. *Ф* and *C*_*i*_/*C*_*a*_ varied between 0.52 and 0.77 (average = 0.59, SD = 0.06) and between 0.30 and 0.70 (average = 0.54, SD = 0.11) respectively. The averages of *C*_*i*_/*C*_*a*_ were higher in the field experiment than in the pot experiment, which was probably due to high rainfall (35 mm higher in 2011 than in 2011 during June–July). The values of *Δ* were higher in the field experiment (average = 7.57‰, SD = 0.18‰) than in the pot experiment (average = 5.84‰, SD = 0.37‰) partly due to higher *C*_*i*_/*C*_*a*_.

### Effect of *N*_*leaf*_ on *Ф* and *C*_*i*_/*C*_*a*_

**Figure 2:**
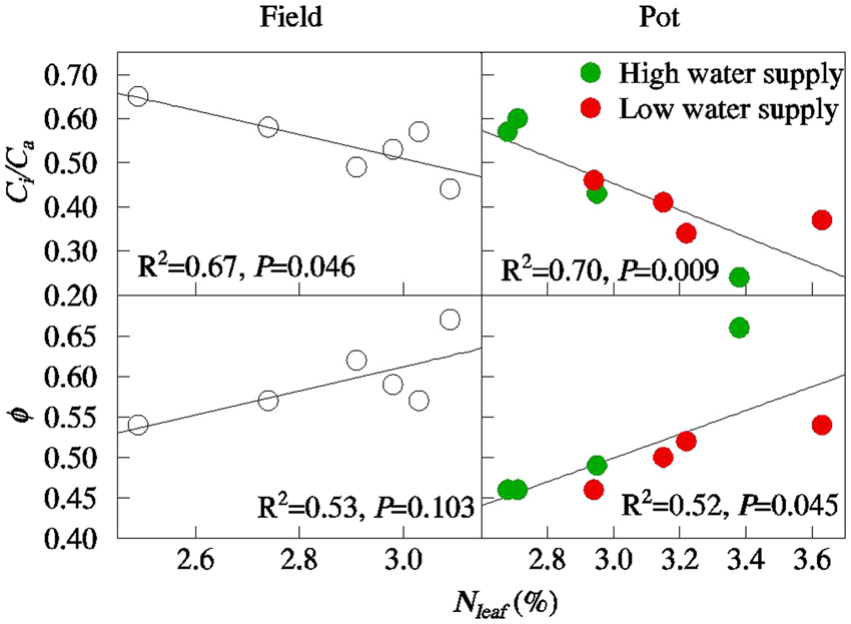
Relationships among leaf nitrogen concentration (*N*_*leaf*_), bundle sheath leakiness (*Ф*), and the ratio of internal and ambient CO_2_ concentrations (*C*_*i*_/*C*_*a*_). Each data point shows the mean of samples taken from the pot experiment (N = 4) or the field experiment (N = 3).

**Figure 3:**
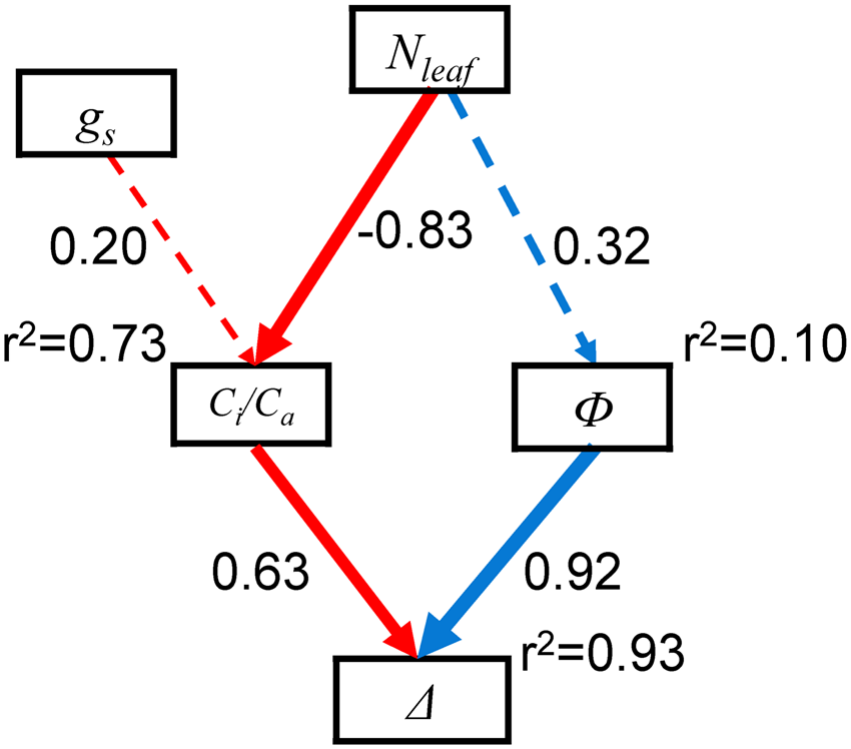
Structural equation modeling (SEM) analysis examining the effects of leaf nitrogen concentration (*N*_*leaf*_) and stomatal conductance (*g*_*s*_) on the ratio of internal and ambient CO_2_ concentrations (*C*_*i*_/*C*_*a*_) and leakiness (*Ф*), and stable carbon isotope discrimination (*Δ*). Square boxes indicate variables included in the model. Results of model fitting: *χ* ^2^ = 6.838, *P* = 0.233, d.f. = 5, N = 14 (Note that high *P*-values associated with *χ* ^2^ tests indicate good model fit to data, i.e., no significant discrepancies). Solid arrows connecting the boxes indicate significant positive and negative effects (*P* < 0.05), respectively; the pathways without significant effects are indicated by broken lines (*P* > 0.05). *r* ^2^ values associated with response variables indicate the proportion of variation explained by relationships with other variables. Values associated with solid arrows represent standardized path coefficients.

*N*_*leaf*_ exerted similar effects on *C*_*i*_/*C*_*a*_ and on *Ф* in the field and pot experiments (Fig. 2). *N*_*leaf*_ was negatively related with *C*_*i*_/*C*_*a*_ (R^2^ = 0.67, *P* = 0.046 in the field experiment; R^2^ = 0.70, *P* = 0.009 in the pot experiment) and was positively related with *Ф* (R^2^ = 0.53, *P* = 0.103 in the field experiment; R^2^ = 0.52, *P* = 0.045 in the pot experiment). SEM models showed that *N*_*leaf*_ had a strong effect on *C*_*i*_/*C*_*a*_ but a slight effect on *Ф* (Fig. 3). Pooling all data, *N*_*leaf*_ explained 73% of the variation in *C*_*i*_/*C*_*a*_ (R^2^ = 0.73, N = 14, *P* < 0.001). *C*_*i*_/*C*_*a*_ decreased by approximately 71% with increasing *N*_*leaf*_ from 2.2% to 4.1%, and *g*_*s*_ had no significant effect on *C*_*i*_/*C*_*a*_.

### Effect of *Ф* and *C*_*i*_/*C*_*a*_ on *Δ*

**Figure 4:**
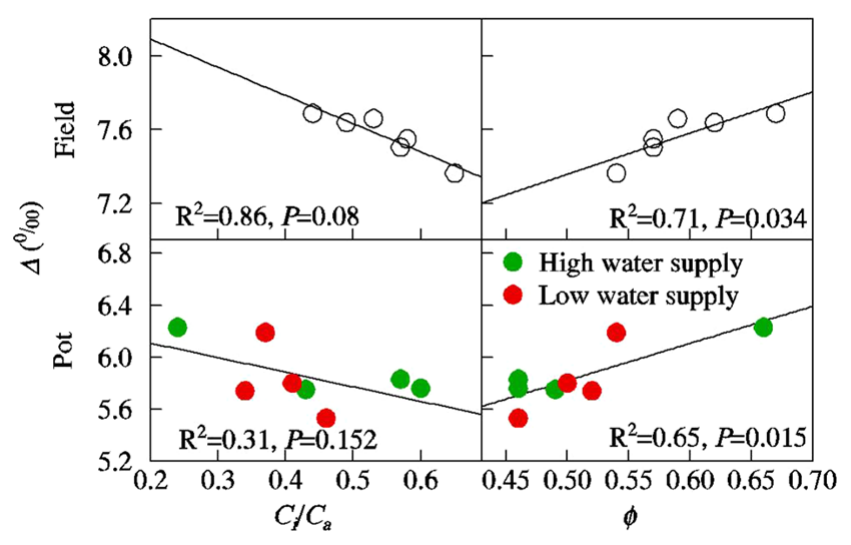
Relationships among stable carbon isotope discrimination (*Δ*), bundle sheath leakiness (*Ф*), and the ratio of internal and ambient CO_2_ concentrations (*C*_*i*_/*C*_*a*_). Each data point shows the mean of samples taken from the pot experiment (N = 4) or the field experiment (N = 3).

*C*_*i*_/*C*_*a*_ was negatively related with *Δ* (R^2^ = 0.86, *P = *0.008 in the field experiment; R^2^ = 0.31, *P* = 0.152 in the pot experiment) while *Ф* was positively related with *Δ* (R^2^ = 0.71, *P* = 0.034 in the field experiment; R^2^ = 0.65, *P* = 0.015 in the pot experiment) (Fig. 4). SEM models further showed that both *Ф* and *C*_*i*_/*C*_*a*_ were important to variations in *Δ* and the contribution of *Ф* was higher than *C*_*i*_/*C*_*a*_ (Fig. 3). *N*_*leaf*_ influenced *Δ* primarily though *C*_*i*_/*C*_*a*_. The effect of *g*_*s*_ on *Δ* was weak.

## Discussion

Our results showed *N*_*leaf*_ had more effect on *C*_*i*_/*C*_*a*_ than *Ф* (Fig. 3). Firstly, the increase in *N*_*leaf*_ strongly decreased *C*_*i*_/*C*_*a*_. *N*_*leaf*_ might stimulate photosynthesis and decrease *C*_*i*_/*C*_*a*_ by increasing the amount of Rubisco and PEPC, or by increasing allocation to photosynthetic organs, such as chloroplasts^24^. Secondly, *N*_*leaf*_ had a slight effect on *Ф*. This species might be able to co-ordinate the activities of Rubisco and PEPC, maintaining a stable Rubisco/PEPC ratio and the difference between dissolved CO_2_ in the bundle sheath and in the mesophyll cells, similar to other NAD-ME subtypes^6^. The stability in *Ф* indicates stable photosynthetic efficiency, which could be one of the physiological reasons why *C. squarrosa* occurs widely in different habitats across the Mongolia grassland.

Our previous survey of *N*_*leaf*_ and *Δ* in *C. squarrosa* showed that *N*_*leaf*_ was negatively correlated with *Δ* across precipitation at the regional scale, the stocking rate in the grazing experiment, and the leaf position within a tiller on the Mongolia Plateau^8^. This study further demonstrated that *N*_*leaf*_ influenced *Δ*, mostly by *C*_*i*_/*C*_*a*_ rather than *Ф* (Fig. 3). However, *Ф* was also important to the variation in *Δ* and should be considered, although it was not influenced by *N*_*leaf*_. Previous studies on C4 plant physiology reported that the *Δ* values in dry leaf were higher in shade conditions due to relatively more leakage of CO_2_ produced during bundle sheath respiration^25–27^. The intensity of shade could be expected to be higher in regions with more precipitation and higher plant biomass^28^, or in areas with higher stocking rate and lower plant cover within the concept of grazing experiment^29^, or for the lowermost leaf within a tiller. Hence, higher *Ф* produced by shade in these places could also contribute to higher *Δ*, which could make the negative relationship between *N*_*leaf*_ and *Δ* stronger. This effect of shade on *Δ* could also had occurred in the field plots with N addition in this study as dominant C3 plants (above 30 cm) were significant taller than *C. squarrosa* (approximately 10 cm).

Two factors can influence the bundle sheath leakiness of C4 plants. One is the difference between dissolved CO_2_ in the bundle sheath and in the mesophyll cells determined by the Rubisco/PEPC ratio, and another is the conductance to leakage determined by a bundle sheath cell wall containing a suberized lamella^11^. The conductance to leakage in *C. squarrosa* was high due to the lack of suberized lamella, which is typical of NAD-ME subtypes^30, 31^. Hence, the value of leakiness should be strongly influenced by the ratio of Rubisco and PEPC activity. Under higher N addition, soil available N could be rich, resulting in higher *N*_*leaf*_ (Fig. 1). Higher *N*_*leaf*_ could contribute to higher *Ф* (Fig. 1) by decreasing the Rubisco/PEPC ratio, which was indirectly confirmed^32^ and supported by previous studies on the negative relationship between leakiness and Rubisco/PEPC ratio^15, 16^. High W supply improved the effect of *N*_*leaf*_ on *Ф* though increasing soil moisture and then plant nitrogen uptake. Meanwhile, *Ф* had no response to W supply, which was not consistent with the result from previous studies showing an increase in *Ф* during drought^7, 33^ or high vapour pressure deficit^32^. Possible reason could be different species or methods. The underlying mechanisms of low W supply on *Ф* though decreasing *C*_*i*_/*C*_*a*_ was still unclear. In terms of the survival of *C. squarrosa* stable *Ф* under low W supply highlights the physiological tolerance.

The extent to which *C*_*i*_/*C*_*a*_ and *Ф* affect *Δ* is a subject or debate. Previous studies reported that *Δ* was mostly influenced by *C*_*i*_/*C*_*a*_ due to variations in *g*_*s*_^34, 35^. Conversely, *C*_*i*_/*C*_*a*_ was primarily controlled by *N*_*leaf*_ not *g*_*s*_ and *Ф* accounted for most of the variations observed in *Δ* in *C. squarrosa* under changing N and W conditions. This phenomenon can also appear in some NADP-ME and PCK subtypes^6, 33, 36^. For instance, *Aristida* spp. deviates from the classical NADP-ME bundle sheath anatomy as it lacks suberized lamellae^37^. Schulze *et al.* ^38^ reported a wide range of *Δ* in *Aristida*. The value of *Δ* was substantially mirrored by changes in *Ф*. Hence, we think that influences of *C*_*i*_/*C*_*a*_ and *Ф* on *Δ* may be species-dependent and vary with different morphological and anatomical characteristics of bundle sheath cells. Also, our results shed light on the effect of *Ф* on *Δ* because *g*_*s*_ were normally regarded as a major cause. More and more studies in recent years reported that *Ф* can be changed by environmental conditions using online measurment or model methods. For instance, high vapour pressure deficit increased *Ф* ^32^; shade reduced *Ф* ^19^. It was demonstrated that the balance of C3 cycle and C4 cycle could be changed by those environmental conditions. Hence, how to predict *Δ* became a challenge.

### Implications

The variation in C4 end-member value in the C3/C4 mixed equation contributes to the uncertainty of estimated C4 percent. Approximately 20 C4 species occur frequently in the Inner Mongolia grassland^39^. More than 70% of them are annual and their growth is opportunistic depending on the frequency and amount of rainfall. The *Δ* value of *C. squarrosa* can represent the majority of vegetation as it is a dominant perennial species in the C4 community across the Inner Mongolia grassland. Pooling the measured values in our study with those from the same region^8^, *C. squarrosa* had a large range in *Δ* from 5.14‰ to 8.90‰. Although the value of *Δ* is relatively stable and independent of soil available N and W or air temperature^3^, the large range of 3.76‰ can cause a bias in the estimated C4 percent. For instance, given that the mean of C3 plants and bulk vegetation is 17‰ and 15‰ respectively^3^, the resulting C4 is 17% when 5.14‰ is used and 25% when 8.90‰ is used. Hence, we suggest that it is better to use a well modeled *Δ* of C4 plants (e.g., geographic distribution based on more sampling) in the C3/C4 mixed equation.

*Ф* is a key parameter which can affect the photosynthetic efficiency of C4 plants and it is extremely difficult to measure it directly. Previous studies reported dry matter *Δ* yield values of 0.31–0.45^10, 11, 13^. The average of *Ф* in *C. squarrosa* was 0.55 although scatted and higher than that reported above, which indicates its lower photosynthetic efficiency in C4 plants. It appears that the photosynthetic performance of this species is closer to C3 plants than other C4 plants such as *Setaria viridis* and *Amaranthus retroflexus*. Relatively low photosynthetic efficiency, together with low height and shallow root depth, could be the major reason for weak competitiveness capacity compared to coexistent C3 plants.

The new technology development, such as tunable diode laser absorption spectroscopy, provides new opportunities for rapidly and concurrently measuring *Δ* and CO_2_ assimilation, which facilitate measurement of photosynthetic *Δ* and calculation of *Ф* ^40^. The advantage of this method compared to dry matter *Δ* is that it is not influenced by post-photosynthesis discrimination^41^. In order to better understand the effect of *Ф* on dry matter *Δ*, more measurements are needed to obtain photosynthetic *Δ*.

## Conclusions

Leaf nitrogen influenced the *Δ* of C4 plants primarily though *C*_*i*_/*C*_*a*_, rather than *Ф*. Both *Ф* and *C*_*i*_/*C*_*a*_ determined *Δ* together and the contribution of *Ф* was stronger. Our study highlights that *Ф* should be well considered in predicting the *Δ* of C4 plants.

## Methods

### Study site

The study was carried out at the Inner Mongolia Grassland Ecosystem Research Station (IMGERS: 43°13′N, 116°14′E), which is located in the Xilin River Basin, Inner Mongolia Autonomous Region of China^42^. The climate in the area is continental temperate semi-arid climate, which is characterized by a cold and dry winter with a warm and moist summer^43^. Long-term (1980–2013) mean annual temperature was 0.9 °C. Long-term mean annual precipitation was 351.4 mm, with 72.8% falling during the growing season (May*–*August). The soil is classified as Calcic-Orthic Aridisol by the U.S. soil classification system. *Stipa grandis* and *Leymus chinensis*, which are domain C3 species, together accounted for >60% of aboveground biomass in the community. *C. squarrosa* is a domain C4 species and normally starts growing at the beginning of June. The annual ambient atmosphere N deposition was <1.0 g N m^−2^ ^9^.

### Pot experiment

To address the first scientific question, we used controlled pot experiment. The pot experiment was carried out at the IMGERS in 2013. To examine the response of *C. squarrosa* to N addition and drought, we used a two-way factorial experimental design. Four N addition treatments were used: control, 10.5, 35.0, and 56.0 g N m^−2^ (as urea) and two W supply treatments: high W supply (ambient rainfall, 160 mm from the beginning of June to the end of July) and low W supply (65% ambient rainfall, 104 mm). Four replicate pots were set for each treatment. We collected seeds from a grassland population near the IMGERS and sowed them in the pots at the beginning of May. Approximately 20 seedlings were sown in one pot (30 cm diameter ×30 cm deep) and received natural rainfall until the end of May. N fertilizers were dissolved in 1 L water, equal to 3.5 mm rainfall, and were then applied to each pot at the beginning of June. The same amount of water was also applied to no N addition plots. A rain shelter was built to prevent ambient rainfall. The amounts of water representing precipitation were 104 mm in June and July and equal amounts of water were applied every 5 days. The water came from a nearby well, with N, phosphorous, and potassium concentrations below detectable levels.

The rain shelter was made of steel pipes, covering 90 m^2^ (6 m × 15 m), and had a slanted roof made of waterproof cloth that could be wound up with a steel roller. The heights of the frame were 50 cm at the south side and 90 cm at the north side respectively. On dry days, the waterproof cloth was rolled to the top of the north side to withdraw the roof from the shelter. When rain was coming, the cloth was rolled back to form the roof of the shelter, and rolled back after rain stopped. Because the shelter was only used during rain events, no significant difference occurred in air moisture and temperature, or light between the inside and outside the shelter. We placed the pots in the soil with a 3-cm edge above the soil surface and the pots at least 1 m away from the edge of each side which followed the suggestions of Heisler-White *et al.* ^44^ and Liu *et al.* ^45^ that a buffer zone of 0.5 m is effective to avoid receiving rainfall. The aisles between treatments pots were 0.4 m.

### Field experiment

To address the second and third questions, we used a long-term field N addition experiment^46^. The field N addition experiment was conducted in a *Leymus chinensis* grassland which had been fenced since 1999 to prevent grazing by large animals. Seven treatments included: control, 0, 5.6, 11.2, 22.4, 39.2, 56.0 g N m^−2^ (added as urea). Each treatment had six replicates. Each plot, except for the control, also received 1.6 g P m^−2^ (as KH_2_PO_4_). The fertilizer mixed with sand applied to the plot surfaces in May during 2006–2011.

### Gas exchange measurement, sample collection and laboratory analysis

The three most mature, fully expanded, and sun-exposed leaves were chosen from each treatment to measure photosynthetic gas exchange parameters at the end of July 2011 (in the field experiment) and at the beginning of August 2013 (in the pot experiment). Gas exchange parameters including photosynthetic rate (*A*), stomatal conductance (*g*_*s*_), and *C*_*i*_ were measured during 08:00–11:30 with an open gas-exchange system (LI-6400, Li-Cor, Lincoln, NE, USA). A 2 × 3 cm^2^ broad leaf chamber with a light source (LI-6400-02B, Li-Cor, Lincoln, NE, USA) was used with the CO_2_ concentration at 400 μmol mol^−1^ and a saturation irradiance of 1500 μmol m^−2^ s^−1^ for the light source. After measuring gas exchange we collected 30 individual leaves from each treatment in both experiments. All samples were dried at 60 °C for 24 hours in a forced-draught oven and homogenized with a ball mill. The carbon isotope composition and N content were then measured using an elemental analyzer (NA 1110; Carlo Erba, Milan) interfaced (ConFlo III; Finnigan MAT, Bremen) with an isotope ratio mass spectrometer (Finnigan MAT253). Carbon isotope data were specified as δ^13^C relative to the Vienna Pee Dee Belemnite standard:

2 $$\delta {}^{13}{\rm{C}}=\frac{{R}_{{\rm{sample}}}}{{R}_{{\rm{standard}}}}-1$$

where *R*_sample_ and *R*_standard_ are the ratios of ^13^C/^12^C in the sample and standard, respectively.

The precision for sample repeats was better than 0.15‰ for δ^13^C and 0.04% for N content in dry matter.

To calculate discrimination, the *δ* ^13^C_air_ values for 2011 and 2013 were obtained from the US National Oceanic and Atmospheric Administration using data from the Ulaan Uul station, which is the closest one located approximately 460 km northwest of IMGERS. Discrimination was calculated as:

3 $$\Delta =\frac{\delta {}^{13}{\rm{C}}{}_{{\rm{air}}}-\delta {}^{13}{\rm{C}}_{{\rm{leaf}}}}{1+\delta {}^{13}{\rm{C}}_{{\rm{leaf}}}}$$

The values of *Ф* were calculated based on *Δ* and *C*_*i*_/*C*_*a*_ using equation (1).

### Statistical analysis

Linear regressions were used to evaluate relationships between *N*_*leaf*_, *Ф*, and *C*_*i*_/*C*_*a*_ and between *C*_*i*_/*C*_*a*_, *Ф*, and *Δ*. Two-way analysis of variance (ANOVA) was used to assess effects of N and W supply on *A*, *g*_*s*_, *C*_*i*_/*C*_*a*_, *N*_*leaf*_, *Ф*, and *Δ*. One-way ANOVA followed by the LSD multiple range tests was used to evaluate the effects of N addition on these response variables under the treatments of ambient precipitation and drought. Structural equation modeling (SEM) was performed to analyze different hypothetical pathways that may explain the effect of *N*_*leaf*_ and *g*_*s*_ on *C*_*i*_/*C*_*a*_ and *Ф* and determine the extent to which *Δ* was influenced by *Ф* and *C*_*i*_/*C*_*a*_. All procedures were carried out in SPSS Version 18.0 (SPSS Inc., Chicago, USA).
